# Assessing patients’ needs in the follow-up after treatment for colorectal cancer—a mixed-method study

**DOI:** 10.1007/s00520-024-08401-w

**Published:** 2024-02-26

**Authors:** Kelly R. Voigt, Esmee A. de Bruijn, Lissa Wullaert, Léon Witteveen, Cornelis Verhoef, Olga Husson, Dirk J. Grünhagen

**Affiliations:** 1 Department of Surgical Oncology and Gastrointestinal Surgery, Erasmus MC Cancer Institute, Doctor Molewaterplein 40, 3015 GD Rotterdam, The Netherlands; 2 On Behalf of Stichting Darmkanker, Utrecht, The Netherlands; 3 Department of Psychosocial Research and Epidemiology, Netherlands Cancer Institute, Amsterdam, The Netherlands

**Keywords:** Follow-up, Colorectal cancer, E-health, Personalised feedback

## Abstract

**Purpose:**

The accessibility of cancer care faces challenges due to the rising prevalence of colorectal cancer (CRC) coupled with a shrinkage of healthcare professionals—known as the double aging phenomenon. To ensure sustainable and patient-centred care, innovative solutions are needed. This study aims to assess the needs of CRC patients regarding their follow-up care.

**Methods:**

This study uses a mixed-method approach divided in three phases. The initial phase involved focus group sessions, followed by semi-structured interviews to identify patients’ needs during follow-up. Open analysis was done to define main themes and needs for patients. In the subsequent quantitative phase, a CRC follow-up needs questionnaire was distributed to patients in the follow-up.

**Results:**

After two focus groups (*n* = 14) and interviews (*n* = 5), this study identified six main themes. Findings underscore the importance of providing assistance in managing both physical and mental challenges associated with cancer. Participants emphasised the need of a designated contact person and an increased focus on addressing psychological distress. Furthermore, patients desire individualised feedback on quality of life questionnaires, and obtaining tailored information. The subsequent questionnaire (*n* = 96) revealed the priority of different needs, with the highest priority being the need for simplified radiology results. A possible approach to address a part of the diverse needs could be the implementation of a platform; nearly 70% of patients expressed interest in the proposed platform.

**Conclusions:**

CRC patients perceive substantial room for improvement of their follow-up care. Findings can help to develop a platform fulfilling the distinct demands of CRC patients during follow-up.

**Supplementary Information:**

The online version contains supplementary material available at 10.1007/s00520-024-08401-w.

## Introduction

Colorectal cancer (CRC), ranking as the third most common type of cancer in the Netherlands, had nearly 12,000 new diagnoses in 2022. Approximately 95% of patients can be treated with surgery, which is considered potentially curative [1]. Despite curative treatment, around 15% of patients develop metastatic disease after resection of CRC [2]. The detection of metastases in an asymptomatic and treatable stage is traditionally the fundament of follow-up. Beyond detection, follow-up care also serves to manage side effects and to provide psychological support when needed [3]. Patients are typically followed up in surveillance protocols for many years after their primary treatment which impacts patients’ lives, their surroundings, and the healthcare accessibility.

With the ageing of the population in general, the increasing incidence of cancer worldwide, and the increase of cancer survivors, it is evident that access to cancer care is under high pressure [4]. The higher number of patients requiring cancer care will receive that care from a decreasing number of healthcare professionals (HCPs): the double aging phenomenon. Cancer care itself has developed enormously and fortunately the number of cancer survivors will continue to increase [5, 6]. Furthermore, shortages of healthcare workers are predicted to triple in the next decade [7, 8], requiring fundamental actions to adapt healthcare systems to be future-proof [9].

An optimal follow-up programme for CRC patients, but in fact all patients, should be patient-centred and adaptive to patients’ needs. There is limited insight into specific patients’ needs and desires regarding a patient-centred follow-up trajectory, and given the increasing influence of digital capabilities, patients’ needs might have changed. Patients in general experience an improved health-related quality of life (HRQoL) when their information needs are fulfilled and when they experience less barriers [10]. During follow-up, the cancer-specific and general health status is measured. The possibility of remote blood withdrawal to measure the cancer-specific biomarkers offers a convenient approach, minimising barriers [11]. Assessing general health is often accomplished through questionnaires. Integrating e-health strategies offers a solution to bridge the gap, delivering tailored support, and nurturing patient empowerment [12, 13]. By analysing the collected data using artificial intelligence techniques, and delivering context-sensitive feedback, a more patient-centric approach can be actualised.

The aim of this study is to assess the needs of CRC patients in the follow-up. This information will provide guidance for clinical practice and will help building an optimal patient-centred digital platform, which satisfies patients’ needs.

## Methods

### Study design

This study is a mixed-method study that is conducted in three phases. To identify the needs of patients in the CRC follow-up, focus group sessions were organised. Next, semi-structured interviews were conducted with patients who were purposefully selected [14]. In the quantitative phase, a questionnaire was sent out to patients in the follow-up after CRC treatment.

### Patient recruitment

Patients curatively treated for CRC were recruited from three hospitals in the Netherlands: two peripheral hospitals (Amphia Hospital, IJsselland Hospital) and one academic hospital (Erasmus Medical Centre). As patients with metastatic disease are faced with other issues compared to primary CRC patients, these patients were excluded from the focus groups and interviews. Eligible patients had to be above the age of 18 and were required to sufficiently speak the Dutch language. Two focus groups were executed inviting six to twelve patients per group, based on considerations of group dynamics and the possibility of no-shows [15].

In total, two focus groups were organised in the Erasmus Medical Centre on two separate evenings in May 2023. Focus group I consisted of participants who have been engaged in a patient-led home-based study for a minimum of 9 months [16]. These participants were reached through postal mail and email. Focus group II comprised of CRC patients in the 3rd–5th year of their follow-up trajectory, who received standard follow-up and attended the outpatient clinic for surveillance. Eligible patients were selected by our research team and subsequently, their oncological surgeon handed them the flyer. The distinction in focus groups was designed to accommodate the different perspectives and anticipations of patients in the primary (patient-led) phase, and those approaching the end of their follow-up journey.

Patients who were interested in participating but who were unable to take part in the focus groups due to factors such as age, travel distance, language barriers, or personal preference were purposefully chosen for the interviews, thereby enhancing inclusivity of the study population [17].

Finally, questionnaires were sent out by email to the remaining patient cohort of the FUTURE-primary study [18], using the Castor platform. Additionally, the questionnaire was also accessible on Stichting Darmkanker, an online Dutch advocacy group serving CRC patients [19].

### Data collection

Details of the data collection can be found in Appendix 1. Prior to the focus groups and interviews, participants completed a questionnaire regarding demographics (Appendix 2). The focus groups were consecutively moderated by at least three of the five researchers, including a female medical student (E.B.), two female medical doctors (K.V. and L.W.), a female epidemiologist (O.H.), and a male surgical oncologist (D.G.).

### Data analysis

The focus groups and interviews were recorded and transcribed verbatim, ensuring the removal of all identifying information. The qualitative data was coded manually using Atlasti_23.1 data software. Open analysis was used to summarise the statements. Multiple quotations could fit into one code if the meaning was the same. To establish themes, subthemes, and code categorisation, axial and selective coding was implemented [20]. Consensus between the two researchers (K.V., E.B) was reached after through in-depth discussion. Relevant quotes from the focus groups and interviews were selected to substantiate the findings. Quantitative data analysis was performed with SPSS Statistics 28.0.1.0 and R version 4.1.2.

Ethical approval for this protocol was obtained from the Medical Ethics Commission (METC) of the Erasmus Medical Centre [MEC-2022–0819]. The commission determined that this study falls outside the scope of the Medical Research Involving Human Subjects Act (Dutch abbreviation: WMO). All participants gave written informed consent for anonymous and confidential use of their data. To report the data and strengthen validity, we used the consolidated criteria for reporting qualitative research (COREQ) checklist (Appendix 7) [21].

## Results

Participant demographics for the focus groups, interviews, and questionnaires are presented in Table 1. A total of 14 patients participated in the focus groups, while 5 patients took part in the interviews. Notably, among the interviewees, two patients had a migration background, one patient had an autism spectrum disorder, and an 84-year-old male participant was also included.

**Table 1:** Baseline characteristics for patients in the qualitative and quantitative study

	Focus group participants, *n* (%)	Interviewees, *n* (%)	Questionnaire respondents, *n* (%)
Total	14	5	96
Sex			
Male	7 (50)	3 (60)	43 (44.8)
Age, median [IQR]	65 [60.8–72.3]	67 [57.5–78]	66.0 [58.0–74.0]
Partner	12 (85.7)	3 (60)	77 (80.2)
Nationality			
Netherlands	14 (100)	3 (60)	95 (99.0)
Other	-	2 (40)	1 (1.0)
Highest educational degree			
Primary school	1 (7.1)	-	4 (4.2)
High school	4 (28.6)	1 (20)	24 (25.0)
Intermediate vocational ed	1 (7.1)	2 (40)	28 (29.2)
Higher vocational ed	8 (57.1)	2 (40)	31 (32.3)
University	-	-	9 (9.4)
Paid job	6 (42.9)	1 (20)	40 (41.7)
Diagnosis			
Screening program	11 (78.6)	2 (40)	34 (35.4)
GP with complaints	3 (21.4)	3 (60)	48 (50.0)
Low hemoglobin	-		6 (6.3)
Other complaints	-		6 (6.3)
Colonoscopy	-		2 (2.1)
Tumour location			
Right-sided colon	3 (21.4)	2 (40)	34 (35.4)
Transverse colon	1 (7.1)	-	9 (9.4)
Left-sided colon	4 (28.6)	1 (20)	15 (15.6)
Rectum	4 (28.6)	2 (40)	28 (29.2)
Unknown	2 (14.3)	-	10 (10.4)
Year of operation			
2014–2020	6 (43.9)	2 (40)	13 (13.5)
2021–2023	8 (57.1)	3 (60)	83 (86.5)
(Neo-)adjuvant treatments			
Chemotherapy	2 (14.3)	-	23 (24.0)
Radiotherapy	-	2 (40)	11 (11.5)
Chemoradiotherapy	1 (7.1)	-	-
Immunotherapy	-	-	2 (2.1)
Colostomy			
Temporary	-	2 (40)	14 (14.6)
Permanent	3 (21.4)	1 (20)	13 (13.5)
None	11 (78.6)	2 (40)	69 (71.9)
Metastases			
Liver	-	-	3 (3.1)
Lungs	-	-	2 (2.1)
Peritoneum	-	-	1 (1.0)
None	14 (100.0)	5 (100)	91 (94.8)
Contact with HCPs			
General practitioner	7 (50)	3 (60)	35 (36.5)
Physiotherapist	5 (35.7)	1 (20)	27 (28.1)
Psychologist	4 (28.6)	1 (20)	12 (12.5)
Dietician	3 (21.4)	1 (20)	24 (25.0)
Nursery care	4 (28.6)	3 (60)	11 (11.5)
Social worker	1 (7.1)	-	6 (6.3)
Other*	3 (21.4)	2 (40)	7 (7.3)
Revalidation	2 (14.3)	1 (20)	2 (2.1)

^*^Other HCPs contacted are the pain specialist (*n* = 2), cardiologist (*n* = 2), clinical geneticist (*n* = 1), rheumatologist (*n* = 1), neurologist (*n* = 1), nephrologist (*n* = 1), pelvic physiotherapist (*n* = 1) lung specialist (*n* = 1), ergo therapist (*n* = 1) and oncologist (*n* = 1)

### Qualitative analysis

The analysis resulted in six major themes and eleven subthemes (Table 2).

**Table 2:** Overview of themes and subthemes

Main themes	Subthemes	Quotes
Cancer and life	Need for help accepting and coping with physical and mental consequences	“Quitting my job was quite a hard process for me that still hurts” (FGI-2)
	Need for help reintegrating in daily life and adapting to a new lifestyle	“It is a strange experience, I really felt having to relearn how to live again. The only thing that occupied my mind during the cancer period was dying.” (FGII-6)
The healthcare system	Importance of a dedicated contact person	“I could always call the ostomy department when I needed them, and then I could always come by for help.” (Int1)
	Need for understanding complications, HCPs, and quicker referral options	“If I had known that there were physiotherapists who specialized in oncology, I might have been able to get there much faster. Yes, now I’m there, and they also recognize the complaints much faster, the fatigue and the pain, and then I think: yes, maybe I didn’t have to have all that sadness.” (FGI-2)
CEA-value	Need for more focus on the psychological aspect	“With the psychological part, my experience was: you have to fix this on your own.” (FGII-3)
	Need for interpreting assistance for the CEA-value	“Well, if you’re going to create such a digital platform, an interpretation of the CEA measurement should of course be included. It can help you calm down when the meaning of the CEA value is available.” (FGI-7)
	Need for anxiety management regarding CEA-value	“Each time you have to undergo a blood test for the CEA-value, it overwhelms my thoughts for the entire week. Every event from that time resurfaces. It would be nice if you can get help for that, so it will become less stressful” (FGI-3)
Quality of life questionnaires	Need for personal feedback through questionnaires, with aid options	“I think, the algorithm in the app will know, sooner than you will know yourself that something is up.” (FGII-4) “It’s helpful if you can see other people’s results, but tricky, because people are unique. For one person this hurts, for another not yet. Everyone is different.” (Int4)
Information provisions	Need for reliable, centered and tailored information	“Yes, the website of the hospital in *** was good, but again those stories are… accountable, so to speak. That also means that a lot of information is taken away, and that in turn makes you feel dissatisfied.” (FGI-6)
	Need for positive stories of other patients	“Afterwards, many people turned out to have had an operation like I had. Famous Dutch people for example, when you hear that… then my situation is not so bad!” (Int5)
Remaining platform issues	Need to take cultural differences, educational levels, security, and regular updates into account	“I think you should make it available for patients to choose their level of information. One might prefer to have precise information up to six decimal places, while another seeks a more generalized overview.” (FGII-4)

### Cancer and life

#### Need for help accepting and coping with physical and mental consequences

Patients encountered symptoms as fatigue, pain, and polyneuropathy during their follow-up, leading to distress. In terms of psychological experiences, patients reported feelings of anxiety, fear for disease recurrence, a sense of vulnerability, and challenges in embracing their ostomy. A subset of patients strongly felt the need to acknowledge these consequences without any complaints. However, accepting these changes was tough, especially when the symptoms affected their daily lives.

#### Need for help reintegrating in daily life and adapting to a new lifestyle

Patients revealed that dealing with the disease often changed their perspective on life. As a result, many felt a strong urge to rethink and reshape their lives, aiming to enjoy life more and adopt healthier habits.

Reconnecting with society after recovering also revealed how the disease affected the people around the patients, like their partners and children. Patients expressed the need for assistance in navigating conversations with their loved ones, because the disease made their relationships more complicated. Besides their family, patients found it hard to chat with others in their daily life. They thought having some assistance in this area would be valuable.

### The healthcare system

#### Importance of a dedicated contact person

The idea prevailed that doctors were busy most of the time, which led patients to seek help from a case manager or ostomy nurse for addressing their questions and concerns. Patients explained that HCPs in such roles exhibited greater interest in their overall well-being. Patients who lacked a designated contact person perceived this as a gap in their follow-up trajectory.

#### Need for understanding complications, HCPs, and quicker referral options

Patients expressed their concern that both physical and mental complications often went unnoticed by doctors, resulting in delays in referrals to other HCPs. Patients often lacked knowledge about symptoms and available treatment. Some patients proposed the idea of a list of common symptoms linked to appropriate HCPs for consultation. Furthermore, they expressed a desire for information about specialised HCPs, such as oncological physiotherapists or specialised dieticians. However, patients emphasised the need for a cautious approach to prevent unnecessary emotional distress.

#### Need for more focus on the psychological aspect

The majority of patients felt that during their follow-up, there was not enough attention for their mental well-being. Several patients faced emotional struggles years after their surgery, and they mentioned that the psychological impact before the surgery was often overlooked. Some suggested having a mandatory session with a psychologist, while others just wanted their tough journey to be acknowledged.

### CEA-value

#### Need for interpreting assistance for the CEA-value

The majority of patients expressed a desire to promptly access their CEA value through their electronic patient files. However, challenges frequently arose when it came to interpreting this value. Variances in the baseline value across different patients further contributed to confusion. Moreover, patients expressed a keen interest in understanding the factors influencing their CEA value and the associated implications.

#### Need for anxiety management regarding CEA-value

The frequent CEA measurements stirred feelings of uncertainty and anxiety, a topic extensively deliberated in the focus groups. For certain patients, this anxiety was momentarily, while others endured persistent anxiety, highlighting the need for assistance in coping with these emotional challenges.

### Quality of Life questionnaires

#### Need for personal feedback through questionnaires, with aid options

The majority of patients were already familiar with completing HRQoL questionnaires, due to their participation in different studies. They acknowledged the value of questionnaires, though some participants found certain questions overly broad and lacking specificity. Many patients missed personal feedback from these questionnaires, expressing curiosity about how their current status compared to previous instances. However, a subset of participants was not sure about such comparisons, particularly if their condition had deteriorated. Nevertheless, they recognised the potential value in receiving feedback as it could provide insights into their overall well-being and serve as an indicator for identifying the need for additional support.

Patients highlighted that initiating contact based on HRQoL questionnaire deviations would yield optimal results when the individual is open and prepared to accept assistance. They believed it was crucial for HCPs to have access to their HRQoL questionnaires to enhance the consultative experience, ensuring that the time spent aligns more effectively with their specific requirements.

### Information provision

#### Need for reliable, centred, and tailored information

Patients commonly felt that as their health complaints increased, so did their need to seek information. A considerable number of patients resorted to online searches for information, expressing a strong preference for reliable sources, a criterion they found difficult to fulfill. They pointed out that the available information was often overly general and designed for a broad population, leaving them in need of more personalised content. As a solution, patients proposed the development of a platform that would allow them to customise the type of information they receive to cater to their specific needs.

#### Need for positive stories of other patients

Several patients drew inspiration and courage from hearing experiences from others, as it put things in perspective. However, it was noted that the preference leaned towards positive stories, as they offered a more optimistic outlook.

### Remaining platform issues

#### Need to take cultural differences, educational levels, security, and regular updates into account

Patient feedback emphasised the necessity to account for cultural differences and varying educational backgrounds when designing a platform. They proposed the inclusion of simplified reading options and suggested that explanatory videos with easy translation options could greatly enhance information comprehension.

Patients also underlined the importance of robust security measures, given the prevailing threat of cyberattacks. Lastly, patients emphasised the need for consistent updates to the platform to ensure accuracy and prevent any potential confusion.

### Questionnaires

The questionnaire was developed using valuable insights gathered from in-depth discussions during the focus groups and interviews. These conversations helped us understand the participants’ perspectives and preferences. With this knowledge, we carefully designed a questionnaire to capture various aspects of their experiences and needs (Appendix 4: Questionnaire).

In total, 96 patients completed the questionnaire. Participants had a median age of 66, were more often female (55.2%). The majority were born in the Netherlands (99.0%), and 50% was diagnosed through the general practitioner due to complaints. Most patients had a partner (80.2%) (Table 1).

### Quantitative analysis

#### Patients’ experiences in the follow-up

**Fig. 1:**
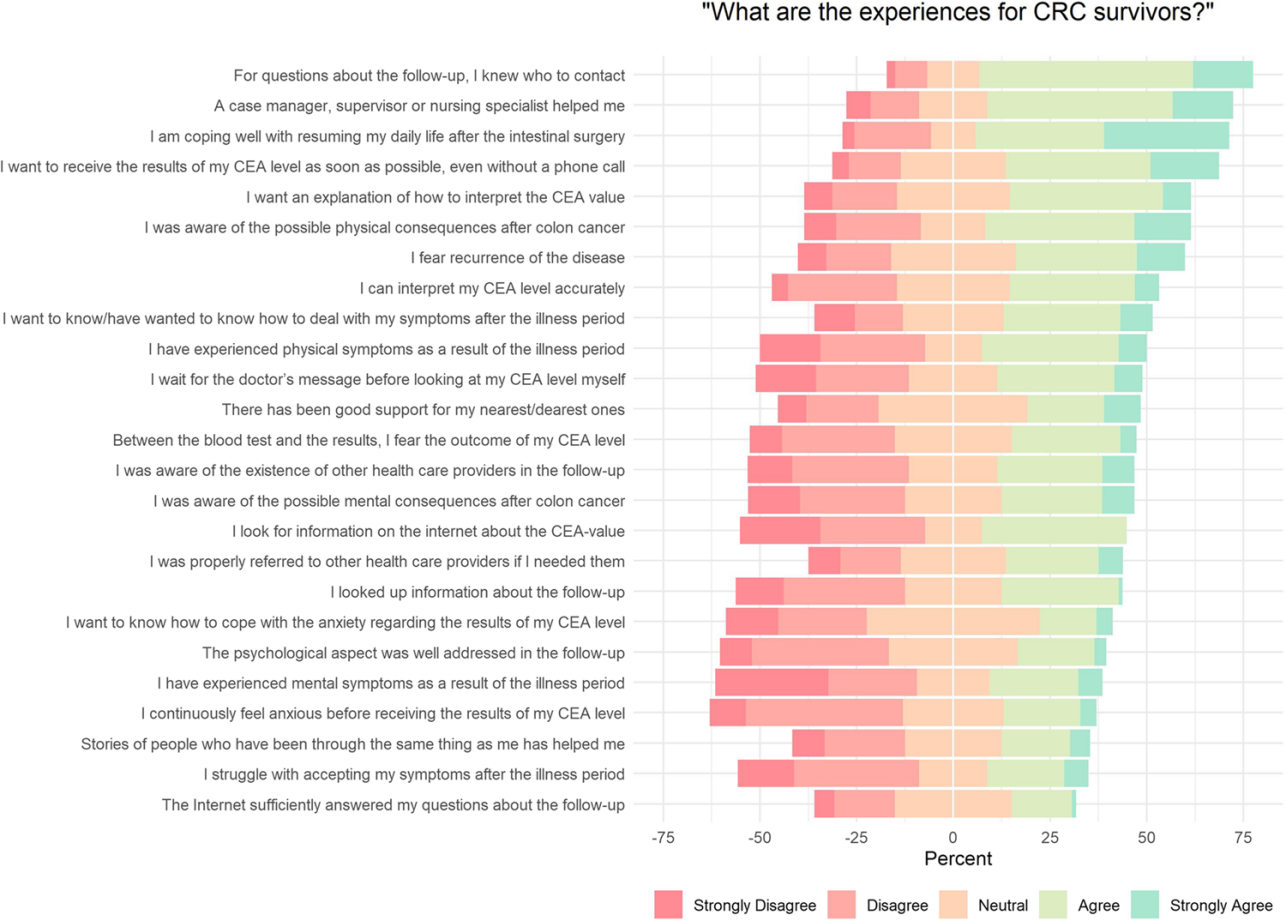
Centred Likert chart of patient experiences during the CRC follow-up

Figure 1 illustrates patients’ experiences during their follow-up. The majority of patients reported being well-informed about whom to contact during their follow-up (70.8%). Interestingly, 40% of patients do not wait for the doctor’s message and proactively check their CEA levels in their online patient file.

#### Patients’ needs in the follow-up

**Fig. 2:**
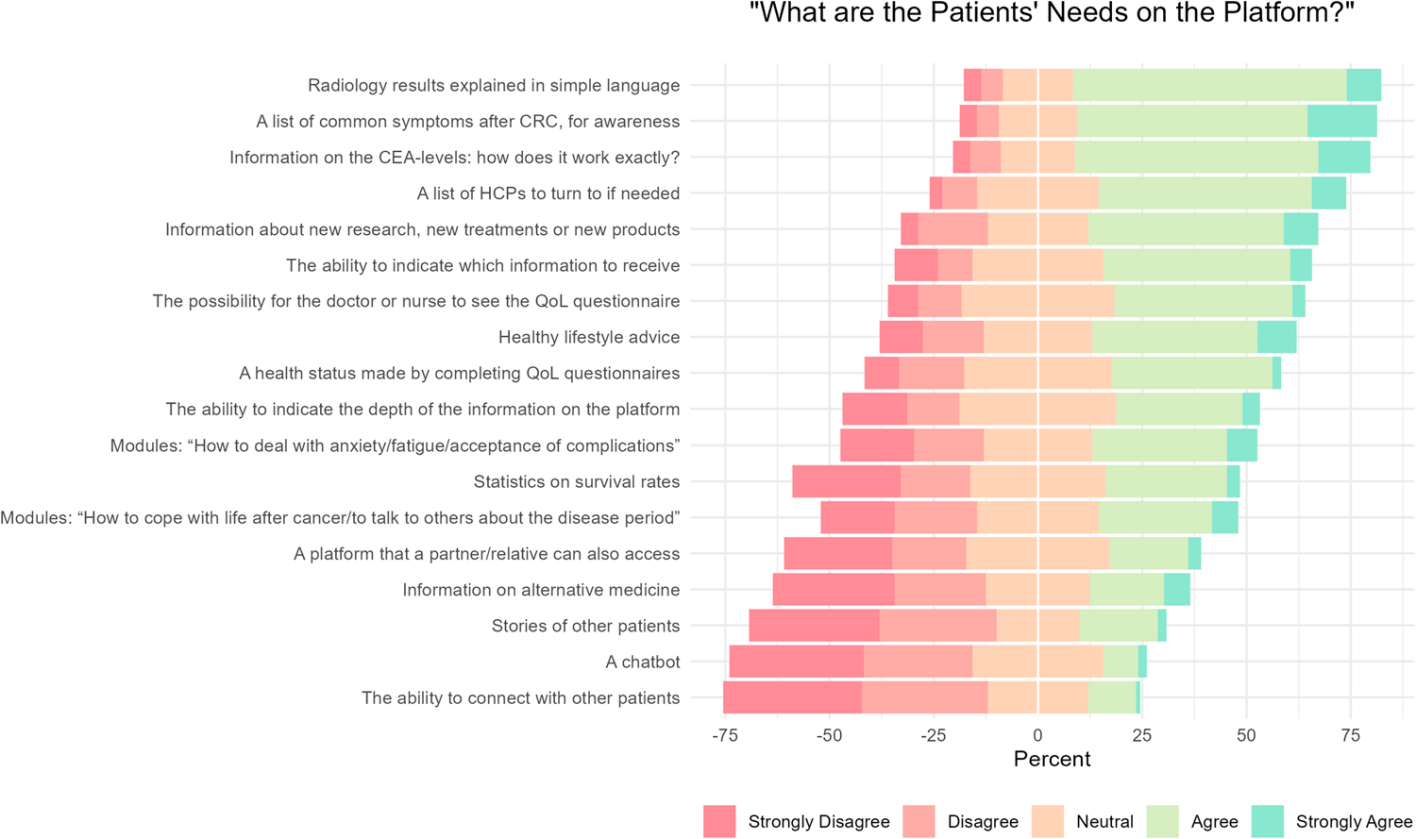
Centred Likert chart of patient needs on the platform

We identified several key areas of concern among patients. The majority prioritised having their radiology results explained in simple terms (74.0%), followed by preferences for a list of common symptoms in the follow-up (71.9%), information concerning CEA-levels (70.8%), and a list of HCPs to consult if necessary (59.4%). Topics for which less patients showed interest were the ability to connect with other patients (12.5%), a chatbot feature (10.4%), and stories of other patients (20.8%) (Fig. 2).

#### A follow-up platform

**Fig. 3:**
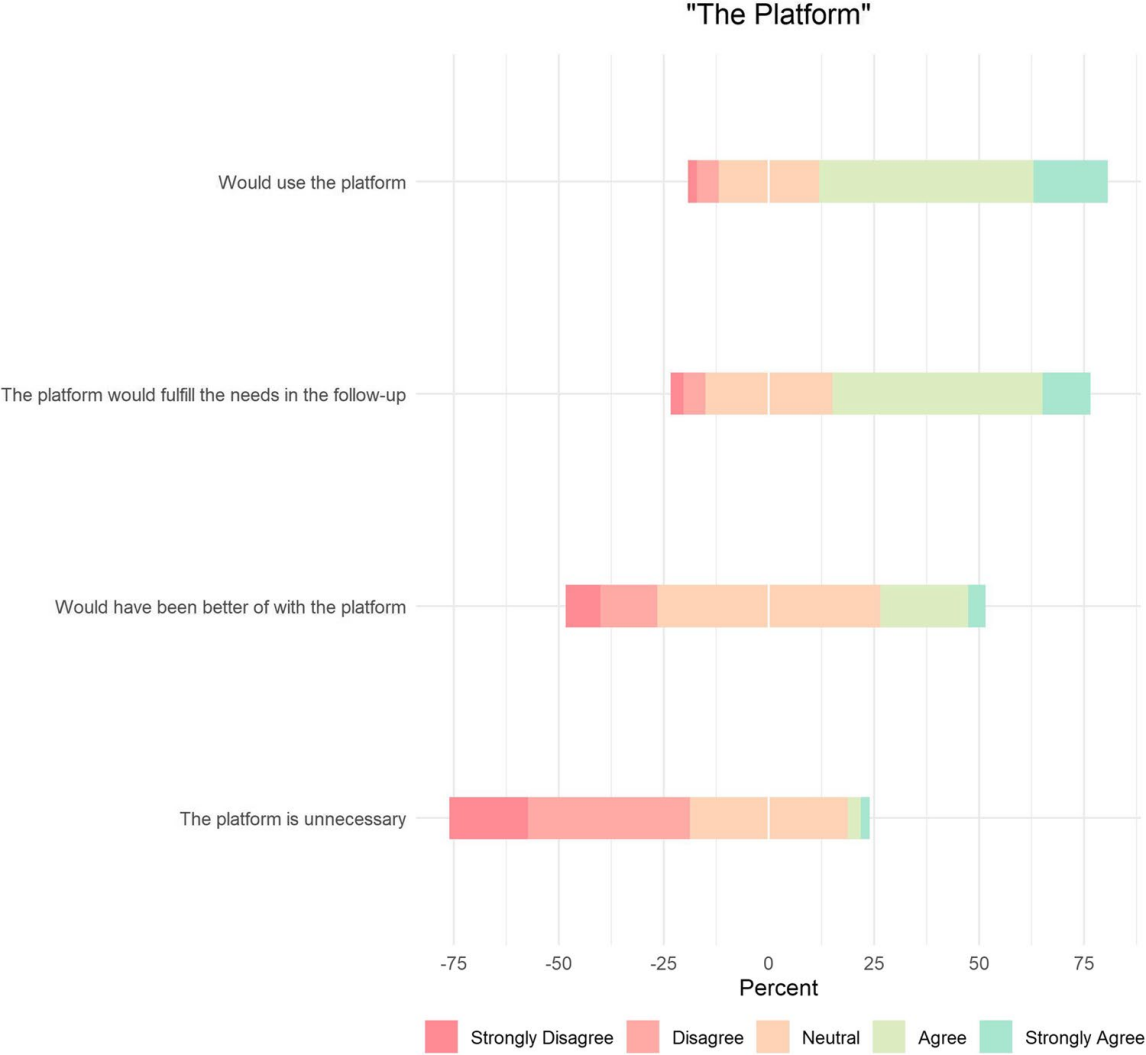
Centred Likert chart of the use of the platform

The majority of patients expressed willingness to use a platform (68.8%). A substantial portion believed that a platform would fulfill their follow-up needs (61.5%). However, opinions were somewhat more reserved regarding whether the platform would make them better off (25.0%). Only a small minority considered the platform unnecessary (5.2%) (Fig. 3).

#### Design of the platform

Patients were asked to outline their ideal platform in their follow-up. A majority favoured receiving their exact CEA value (64.6%), with the option of ‘good’ or ‘not good’ being popular as well (40.6%). In the case of elevated CEA results, a vast majority still preferred discussing with their doctor before scheduling a CT scan (81.3%).

Considering HRQoL questionnaire results, most patients indicated a preference for comparison with their previous questionnaires (67.7%), followed by peers (37.5%), and then the healthy Dutch population (7.3%). If HRQoL results indicated poor outcomes, half of the patients would initiate contact with an HCP (52.1%), while the other half preferred their HCP to reach out to them (47.9%). More detailed information regarding the design of the platform is summarised in Appendix 6.

## Discussion

This mixed-method study examined the needs of CRC patients during their follow-up. Various needs were mentioned by participants, some even multiple times, as highlighted by a participant of the focus groups “Everybody is different, but our complaints are not unique” (FG I-7)*.* From the perspective of patients, there are enough opportunities to improve and tailor the follow-up.

A possible approach to address a substantial part of the diverse needs could be the implementation of a platform. Through such a platform, patients could access personalised resources, and receive updates about their cancer specific and general health status during their follow-up care. HCPs could also use the platform to monitor patients’ progress, and offer guidance. In order to measure and prioritise the specific needs of CRC patients and evaluate the impact of the platform, we incorporated this concept into the questionnaire. By including questions that directly addressed the usage of the platform, we could gather valuable insights into how the platform influenced patients’ needs.

Several patients brought up mental health issues during their CRC follow-up. These concerns consisted of anxiety for recurrent disease and feelings of vulnerability following their hospitalisation. For example, the use of artificial intelligence could help assess the outcomes of these questionnaires; patients could be directed to use a e-Health self-management application when they experience mild symptoms [22]. When patients have severe complaints, an advice to contact a HCP can be sent to patients. This proactive approach can play a role in patient empowerment but also potentially reducing the pressure on the healthcare system. According to the data collected from the questionnaire, almost 40% of the participants expressed a desire for modules that focus on managing feelings such as anxiety.

In the context of addressing patients’ needs, it is important to acknowledge the anxiety that can arise around CEA test results. Almost a quarter of the patients feel anxious before receiving the CEA results. This was mentioned several times by participants. To mitigate this anxiety, an approach could involve communicating the test results shortly after they are obtained by immediate phone notifications or by displaying the results directly on the platform. Moreover, the platform could also play a role in identifying patients who are struggling with excessive anxiety related to these results. For such individuals, the platform could offer specialised support and resources tailored to their emotional well-being. This comprehensive approach underscores the platform’s potential not only to provide medical information but also to cater to the psychological and emotional needs of CRC patients throughout their follow-up journey [23].

It is evident that there is a significant desire for a designated contact person who patients can rely on. This contact person could be integrated into the platform’s functionality, offering patients a direct channel to seek guidance, clarification, or assistance. However, it is also crucial to consider the potential impact of such a contact point on an already strained healthcare system, struggling with a shortage of personnel [24]. The platform could explore the use of a chatbot, which serves as an automated assistant. A chatbot could respond to frequently asked questions and provide immediate support, thus minimising the impact on the healthcare system’s resources [25].

These insights have shed light on a shared concern among CRC patients: the lack of sufficient and appropriate information. By collaborating with patient associations, the platform could create an overview of the necessary resources, ensuring that patients receive accurate information tailored to their needs. A critical aspect is finding a balance between information overload and insufficient guidance. Recognising the diverse information preferences of patients, offering them personalised information on the platform is a thoughtful approach. This way, patients can opt for the level of information that aligns with their needs and preferences, improving their engagement with their own follow-up trajectory.

A systematic review describing unmet needs of CRC survivors came to a parallel conclusion, emphasising the requirement for information regarding symptom expectations and management, as well as the continuous support throughout the follow-up [26]. A recent study conducted three focus groups with the primary objectives to gather insights from CRC survivors to improve care, and explore the potential of e-health technology [27]. The themes from these focus groups align with our own findings, highlighting the pressing need for information on symptom expectations and management, as well as sustained support during follow-up. These similarities not only validate our research, but also raise awareness for targeted interventions which address these needs and improve the overall well-being of CRC patients during their follow-up care.

The perspective of CRC HCPs on e-health applications within the CRC care pathway has already been examined through interviews [28]. The opportunity to use online information services about treatment options and common side effects were mentioned. However, some concerns were also raised regarding the inability to use e-health by specific patient groups. A comprehensive understanding of the extent to which patient groups encounter challenges in using e-health applications will become more evident as further studies are conducted in this domain. To ensure the platform’s effectiveness and user-friendliness, a dynamic approach is needed. By consistently seeking feedback from patients, the platform’s design and functionality can be fine-tuned to match evolving needs and preferences, which can lead to a more efficient and targeted follow-up care. This contributes to the challenges posed by the double ageing phenomenon.

### Suggested platform

**Fig. 4:**
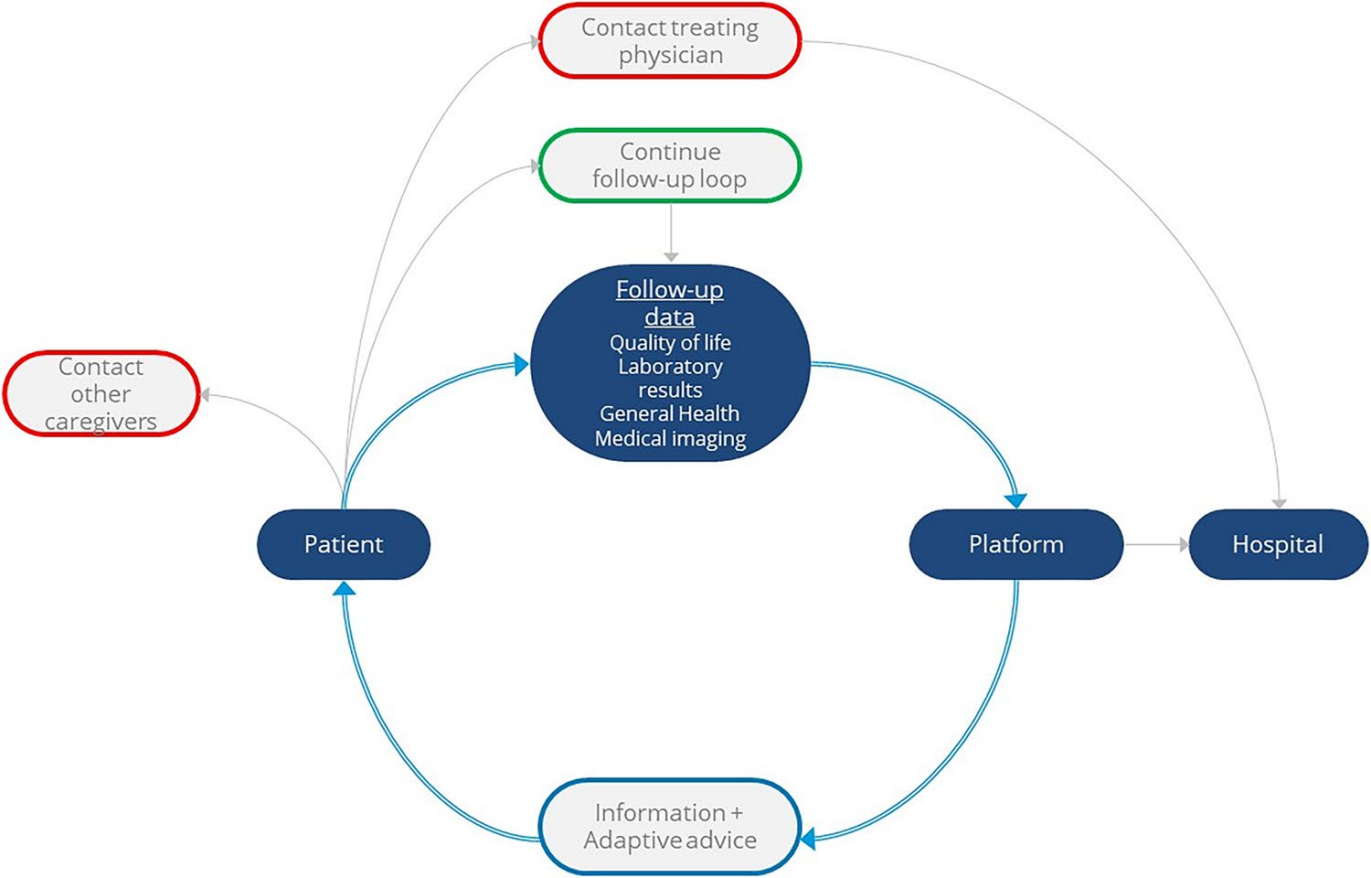
Schematic representation of the proposed platform

The platform will be tailored to support patients’ evolving needs during their follow-up. This is especially important during periods of high pressure on cancer care accessibility, such as the COVID-19 pandemic, and in the upcoming years with the expected growing number of cancer survivors compared to the number of healthcare providers. The use of electronic Patient Reported Outcome Measures (ePROMs) will ensure a comprehensive understanding of patients’ well-being [29]. On the platform, patients will be able to receive immediate feedback and contextual information regarding their ePROMs (over time, and in comparison to peers), laboratory results, and medical imaging reports. With the help of thresholds for ePROMs, the platform could offer specialised support and advise referrals for patients facing problems such as anxiety or psychosocial challenges based on their ePROMs [30, 31]. A schematic overview of this feedback loop for the platform is shown in Fig. 4. For a comprehensive evaluation of the platforms’ effectiveness, an implementation study is essential, enabling continuous assessment and refinement aligned with the evolving needs of both patients and healthcare providers. To assess the effectiveness of the platform, health-related quality of life questionnaires of patients using the platform and those undergoing traditional follow-up should be compared. Some of the potential constraints of the platform are related to patient, clinical, and resource workflows. The impact on the workload of caregivers due to automatic referrals for patients experiencing deterioration, information overload for patients, and efficient workflows should be assessed before large implementation [29, 31].

## Limitations

Despite the explorative nature of this study, data saturation could not be reached due to the limited sessions. In response, an open question was incorporated to encourage respondents to provide extra insights. However, the extent of input obtained was limited. Another limitation relates to potential selection bias. The majority of participants were involved in a clinical study or were recruited via networks of ‘Stichting Darmkanker’. This could imply a predisposition toward higher engagement and interest in self-managing their condition. Importantly, it should be recognised that these individuals represent the intended user group for a potential platform. To ensure inclusivity, one focus group was specifically convened with patients from the outpatient clinic. Additionally, interviews were conducted with patients who might be less inclined to express their opinions within a group, thereby catering to a broader spectrum of perspectives.

## Conclusion

While CRC patients in the follow-up have different experiences, their needs are alike. A personalised platform holds potential to fulfill those needs, when it focuses on different aspects of the follow-up. These insights can help to develop a platform catering to the distinct demands of CRC patients during follow-up. Continuous evaluation should determine whether a platform meets patients’ needs.

### Supplementary Information

Below is the link to the electronic supplementary material.

Supplementary file1 (PDF 490 KB)

Supplementary file2 (DOCX 30 KB)

## Data Availability

The datasets generated during and analysed during the current study are available from the corresponding author on reasonable request.
