# Supplementary material for: Assessing patients’ needs in the follow-up after treatment for colorectal cancer—a mixed-method study
Source: Support Care Cancer. 2024 Feb 26;32(3):192. doi: 10.1007/s00520-024-08401-w (PMC10896820; doi:10.1007/s00520-024-08401-w)
Supplement: Supplementary file 2 — Supplementary file2 (DOCX 30 KB) [file 520_2024_8401_MOESM2_ESM.docx]

**Assessing patients’ needs in the follow-up after treatment for colorectal cancer - a mixed-method study**

Kelly R. Voigt^1^, Esmee A. de Bruijn^1^, Lissa Wullaert^1^, Léon Witteveen^2^, Cornelis Verhoef^1^, Olga Husson^1,3^, Dirk J. Grünhagen^1^
^1^Erasmus MC Cancer Institute, Department of Surgical Oncology and Gastrointestinal Surgery, Rotterdam, Netherlands.
^2^On behalf of Stichting Darmkanker, Utrecht, the Netherlands
^3^Department of Psychosocial Research and Epidemiology, Netherlands Cancer Institute, Amsterdam, The Netherlands

**Corresponding author**

Dr. Dirk J. Grünhagen

Email: d.grunhagen@erasmusmc.nl | Tel.: +31 10 7042125

Department of Surgical Oncology and Gastrointestinal Surgery

Erasmus MC Cancer Institute

Doctor Molewaterplein 40, 3015 GD Rotterdam, The Netherlands

**Supplementary materials**

Appendix 1: Data collection details

Appendix 2: Sociodemographic questions prior to the focus groups, interviews and questionnaire

Appendix 3: Focus group script

Appendix 4: Interview script

Appendix 5: Specific CRC-follow-up needs questionnaire

Appendix 6: Outcomes of design of the platform

Appendix 7: COREQ Checklist

**Appendix 1**

*Data collection*

One researcher (E.B, L.W., or K.V.) took notes during the focus groups and interviews. Before the start, the study team introduced the study, following a concise overview. A script was made beforehand with two main questions: 1. “What did you miss in the follow-up after the colorectal cancer treatment?” and 2. “What would you like to see if a digital platform becomes available regarding the follow-up of colorectal cancer?” (Appendix 3). The moderator facilitated participant interactions. The conversation flowed naturally, complemented by supplementary inquiries when deemed necessary [20]. Both focus groups lasted 2 hours.

After the focus groups, an interview format was created based on the insights from the focus group responses (Appendix 4). Depending on the preference of the patient, the interview took place either at the hospital or over the phone. EdB and KV took turns in leading the conversation, the other one took notes. The interviews lasted a maximum of 60 minutes. All participants in the focus groups and interviews received travel allowance, parking fees and a €25 gift voucher.

Using the qualitative data, an online specific CRC-follow-up needs questionnaire was formed in Castor EDC (Appendix 5). This method was chosen for prioritizing different needs. After 10 days, a reminder was sent to the participants receiving the questionnaire through e-mail. The form closed after 3 weeks of publication.

**Appendix 2:** Sociodemographic questions prior to the focus groups, interviews and questionnaire

| Appendix 2: Sociodemographic questions prior to the focus groups, interviews and questionnaire |
| --- |
| How did you receive the invitation for this questionnaire?  What is your sex?  What is your age?  What is your partner status?  What is your nationality?  What is your highest level of education?  Do you have a paid job?  What are your hobbies?  In which year did you have the operation?  How did the CRC trajectory start (; went to the doctor because of complaints or joined the screening program?)  Where was the tumor located?  Do you/did you have a colostomy?  Besides the operation, did you receive radiotherapy or chemotherapy?  Do you/did you have metastases?  With which caregivers did you have contact after the operation?  Did you revalidate in a revalidate center after the operation? |

**Appendix 3:** Focus group script

| Introductory question | Would you like to introduce yourself? |
| --- | --- |
| Questions | - Who are you - What do you do in your daily life? - How long ago was your operation? - Can you summarize how it is going now? - Who/what brings color to your life? |
| Main question number 1 | **What did you miss in the follow-up after the colorectal cancer treatment?** |
| Sub questions | - How did the follow-up go for you? - Which experiences were positive? - Did you search for information on the internet? Could you find the information you were looking for? - Who was your point of contact in the follow-up? - Did you experience seeing your CEA-value in your online patient file without knowing how to interpret the value? - Were you in need of more contact with your health care provider? If so, how easy was it for you to arrange this? - Did you feel that your practitioner had enough time for you on the moments you needed it? |
| Main question number 2 | **What would you like to see if an digital platform becomes available regarding the follow-up of colorectal cancer?** |
| Sub questions | - What do you think of the personal feedback system? - How do you envision it when your CEA-value is elevated and you need to make a CT-scan; do you want to talk to the doctor first or would you go to the scan directly? - What needs to happen when it turns out, after you filled in a QoL questionnaire, it turns out that your emotional wellbeing is declining; would you contact the general practitioner yourself? Would you contact the hospital? Does the hospital have to contact you? - Besides the platform, what would help you in the follow-up? - Would you like to know how you function compared with peers? - What kind of information would you like to see on the platform? - If we would say that there is a solution for anxiety or tiredness, would you want help/information about that? - Do you need an overview of your disease? - Would you like to see your information in text or video/audio? - Would you like it if your partner can go to the platform as well? - What do you think about privacy? Is your doctor allowed to see your information on the platform? |
| Concluding questions | **Summary** |
| Question | - Did you miss anything? Is there something else you would like to say? |

**Appendix 4:** Interview script

| Introductory question | Would you like to introduce yourself? |
| --- | --- |
| Questions | - Who are you - What do you do in your daily life? - How long ago was your operation? - Can you summarize how it is going now? - Who/what brings color to your life? |
| Open question | **Did you miss anything in the follow-up? How can we improve this using the platform?** |
|  |  |
| CEA-value | ***Right now, you go to the hospital for blood withdrawal for the CEA-value. As we speak, research is being done for doing this more from home. The CEA-value will appear on the platform with interpretation and personal feedback.*** |
| Questions | - Did you miss this? - How do you see the personal feedback? - Would you like to see this with the true value, or just if it is good or bad? (colors, smileys?) - Imagine: your CEA-value is elevated; do you want your doctor to contact you or do you want to plan a CT-scan through the system, talking to you doctor after making the CT-scan? - Do you have other ideas of how we can handle this? |
| Quality of Life questionnaires | ***You might already fill in QoL questionnaires for other studies. Ultimately, we would like those questionnaires on the platform, probably around the time you are also drawing blood for your CEA-value. With the modern technologies we can compare your outcomes with previous outcomes. With that comparison, we can make a rapport/advice, based on your personal situation.*** |
| Questions | - How do you see the QoL questionnaires? Which topics should be covered? - What needs to happen when it turns out, after you filled in a QoL questionnaire, it turns out that your emotional wellbeing is declining; would you contact the general practitioner yourself? Would you contact the hospital? Does the hospital have to contact you? - How do you think about statistics/a rapport on your wellbeing? - Do you want to be compared with peers? - Do you want to be compared with the ‘healthy’ Dutch population? - Do you have other ideas of how we can handle this? |
| Information provision | ***Right now, there is a lot of information on different websites about the follow-up of CRC. Sometimes, the hospital will give you a flyer, or you can look for information on their website, but it is mostly wide spread. We would like to unite the information on the platform.*** |
| Questions | - How do you see the information? - Do you need ‘psychosocial’ tips, for example: “How do I deal with …” (anxiety, tiredness, fear for my CEA-value, talking with my partner about this) - Do you envision a list with common complaints in the follow-up, so you can recognize them and find help? - Do you want a list with healthcare professionals who you can turn to when needed? - Would you like to see patient stories? - Would you like to see updates of scientific research about the follow-up? - What about tips for healthy living / patient tips? - Do you need the information to be adaptable on your personal situation (for example: I do not have a colostomy so I do not need information about that subject) - How would you like to receive the information? In text, video, audio, in English? - Is it important for you that you can decide on which level you will receive the information? (6 decimal places or short) - Do you have other ideas of how we can handle this? |
|  | *Summary* |
| Question | - Did you miss anything? Is there something else you would like to say? |

**Appendix 5:** Specific CRC-follow-up needs questionnaire

**Part 1:** Sociodemographics (Supplementary table 2)

| **1**  Strongly disagree | **2**  Disagree | **3**  Neutral | **4**  Agree | **5**  Strongly agree |
| --- | --- | --- | --- | --- |

**Part 2:** Experiences - rank the statements below

| 1 | I am coping well with resuming my daily life after the colorectal cancer surgery |
| --- | --- |
| 2 | I have experienced physical symptoms as a result of the illness period (such as fatigue, stoma-related issues, polyneuropathy, pain, etc…) |
| 3 | I was aware of the possible physical consequences after colorectal cancer |
| 4 | I have experienced mental symptoms as a result of the illness period (such as sadness, vulnerability, anxiety, etc…) |
| 5 | I was aware of the possible mental consequences after colorectal cancer |
| 6 | I struggle with accepting my symptoms after the illness period |
| 7 | I want to know/have wanted to know how to deal with my symptoms after the illness period |
| 8 | I fear recurrence of the disease |
| 9 | I continuously feel anxious before receiving the results of my CEA level |
| 10 | Between the blood test and the results, I fear the outcome of my CEA level |
| 11 | I want to know how to cope with the anxiety regarding the results of my CEA level |
| 12 | I want to receive the results of my CEA level as soon as possible, even without a phone call from the doctor |
| 13 | I wait for the doctor’s message before looking at the CEA result myself |
| 14 | I can interpret my CEA result accurately |
| 15 | I look for information on the internet about the CEA value |
| 16 | I want an explanation of how to interpret the CEA value |
| 17 | The psychological aspect was well addressed in the follow-up |
| 18 | I was aware of the existence of other health care providers in the follow-up (e.g. oncological physical therapist, podiatrist for neuropathy, psychologist, etc…) |
| 19 | I was properly referred to other health care providers (e.g. oncological physical therapist, podiatrist for neuropathy, psychologist, etc…) if I needed them |
| 20 | For questions about the follow-up, I knew who to contact |
| 21 | A case manager, supervisor or nursing specialist helped me (or would help me) |
| 22 | I looked up information about the follow-up |
| 23 | The internet sufficiently answered my questions about the follow-up |
| 24 | Stories of people who have been through the same thing as me has helped me (or would help me) |
| 25 | There has been good support for my nearest/dearest ones |

**Part three:** Needs on the platform – rank the statements below

| **1**  Really don’t need | **2**  Don’t need | **3**  Neutral | **4**  Need | **5**  Really need |
| --- | --- | --- | --- | --- |

| 1 | Understanding CEA levels: explanation of how CEA levels work |
| --- | --- |
| 2 | My ultrasound, MRI and X-ray results explained in simple language |
| 3 | A list of common symptoms after colorectal cancer, so you can be aware of them |
| 4 | A list of healthcare providers you can turn to if needed |
| 5 | Statistics regarding survival rates |
| 6 | Lifestyle advice for a healthier life |
| 7 | A health status made by completing quality of life questionnaires |
| 8 | Information on forms of alternative medicine (acupuncture and the like…) |
| 9 | Stories of other patients |
| 10 | Modules: “How do I deal with anxiety” or “How do I deal with fatigue” or “How do I deal with acceptance of the effects of the disease” |
| 11 | Modules: “How do I cope with life after cancer” or “How do I talk to others about the disease period” |
| 12 | News updates on new research, treatments, and products related to cancer |
| 13 | A chatbot (“talk”function with the computer) that answers my questions immediately |
| 14 | Connect with other patients for support and information sharing |
| 15 | A platform accessible to your partner or relative |
| 16 | The ability to customise the information you receive |
| 17 | Specify the depth of information you want on the platform |
| 18 | The possibility for my doctor or nurse to see my quality of life questionnaires |

**Part four:** Design of the platform

1. I want to receive my CEA-value in this way (multiple options available):

- The value itself (for example 3.2)
- Good/not good
- With colors
- With smileys
- Different: …

1. Imagine your CEA value has increased, indicating a potential concern. You notice this on the platform, and the next course of action is to undergo a CT-scan to investigate the possibility of cancer recurrence. How would you prefer to receive information and support during this process?

- I looked at my CEA-value on the platform. I immediately plan a CT-scan myself through the platform and will talk to my doctor later
- I want a (telephone) consult with my doctor before I make the CT-scan, and talk to the doctor again afterwards

1. Together with the report of my quality of life questionnaire, I would like to see how…:

- I’m doing compared to the last time/times
- I’m doing compared to peers
- I’m doing compared to ‘healthy’ people in the Netherlands

1. When, through the quality of life questionnaires, it turns out I’m doing worse, and the platform advices me to contact a healthcare professional, then: …

- I will contact the advised health care professional (for example the general practitioner or the physiotherapist) who will help me
- I want the hospital to call me and ask what I need
- I will do something else: ….

1. I want to receive my information through: … (multiple options available)

- Text
- Video
- Audio
- Pictures

1. How would you like to reach the platform?

- Through an app on my phone
- Through a website
- Both

**Part five:** Use of the platform

| **1**  Strongly disagree | **2**  Disagree | **3**  Neutral | **4**  Agree | **5**  Strongly agree |
| --- | --- | --- | --- | --- |

| 1 | I would use the platform |
| --- | --- |
| 2 | The platform would fulfill my needs in the follow-up |
| 3 | If I could have used the platform in my follow-up, I would have been better off |
| 4 | I think the platform is unnecessary |
| If you have suggestions for the upcoming platform, please let us know below:  …...... | |

**Appendix 6:** Outcomes of design of the platform

| Appendix 6: Percentages of design of the platform | |
| --- | --- |
| 1. How do you want to receive the CEA-value? | The value itself (64.6%)  Good/not good (40.6%)  Colors (2.1%)  Smileys (1.0%) |
| 1. What is your action when the CEA-value is elevated? | First a consult with the doctor, then CT-scan (81.3%)  Immediately to the CT-scan (18.7%) |
| 1. To whom do you want to compare your quality of life questionnaire results? | Yourself (67.7%)  Peers (37.5%)  The normal Dutch population (7.3%) |
| 1. What would you do if you discover that your quality of life assessment indicates that you're experiencing a decline? | Would contact HCPs him/herself (52.1%)  Reach out to the hospital (47.9%) |
| 1. How would you like to receive your information on the platform? | Text (95.8%)  Video (7.3%)  Audio (2.1%)  Pictures (6.3%) |
| 1. How do you want to access the platform? | Phone (16.7%)  Website (24.0%)  Both (59.4%) |
